# Supplementary material for: Possible existence of optical communication channels in the brain
Source: Sci Rep. 2016 Nov 7;6:36508. doi: 10.1038/srep36508 (PMC5098150; doi:10.1038/srep36508)
Supplement: Supplementary Information [file srep36508-s1.pdf]

Supplementary Information for "Possible existence of optical communication channels in the brain"

Sourabh Kumar,[1] Kristine Boone,[1] Jack Tuszyński,[2,3] Paul Barclay,[1,4] and Christoph Simon[1]

[1]*Institute for Quantum Science and Technology and Department of Physics and Astronomy, University of Calgary, Calgary T2N 1N4, Alberta, Canada*
[2]*Department of Oncology, University of Alberta, Cross Cancer Institute, Edmonton T6G 1Z2, Alberta, Canada*
[3]*Department of Physics, University of Alberta, Edmonton T6G 2E1, Alberta, Canada*
[4]*National Institute for Nanotechnology, Edmonton T6G 2M9, Alberta, Canada*

Approximate analytic expressions for the guided modes. The exact analytic form for the guided modes of cylindrical myelinated axons with the unconventional fiber geometry (the refractive index of the cladding higher than the core) would involve linear combinations of different Bessel functions, similar to those in [S1]. However, we can come up with much simpler approximate expressions of the mode profiles observing those generated by the software. The cylindrically symmetric modes used in our simulations (identical to Fig. 1b–e from the main text) have a radial intensity dependence that is very close to a Gaussian, with peak intensity at the center of the myelin sheath, and with continuously decreasing intensity on both sides. The beam diameter corresponds to some fraction of the thickness of the myelin sheath (intensity of the form $Ae^{-(r-r_0)^2/(2\sigma^2)}$, where A, r, r_0, and 4σ are the amplitude, radial coordinate, the radial distance of the center of the myelin sheath, and the beam diameter respectively). The fraction can be estimated by knowing the fraction of the optical power inside the myelin sheath (e.g. 95.4 % power in the myelin would imply that $4\sigma = d$, where d is the myelin sheath thickness). Note that this discussion about the approximate Gaussian shape of the field intensity is just to provide an intuition about the modes. In all our simulations, we use the modes directly generated by the software, and not the ones based on these simple approximate expressions.

In Supp. Fig. 1, we tabulate the modal fraction (fraction of the total power of a mode) inside the myelin sheath for different axon calibers and different wavelengths to illustrate their confinement. The power confined in the myelin sheath varies from 99.58 % for the best confined mode in the thickest axon in our simulations to 82.13 % for the least confined mode in the thinnest one, which is still higher than the typical confinement in the core of practical single mode fibers [S2] used for communication over tens of kilometres. Good confinement is necessary to limit interactions with the inhomogeneous medium inside and outside the axon. The scatterers inside the axon are the cell organelles, e.g. mitochondria, microtubules, and neurofilaments, whereas on the outside there are different types of cells, e.g. microglia, and astrocytes. There are guided modes with much weaker power confinement in the myelin sheath (less than 50 %). However they might soon be lost to the inhomogeneities, and are therefore neglected. Supp. Fig. 1 also explicitly lists the thickness of the myelin sheath (d), the longest permissible wavelength (λ_{max}), the wavelength corresponding to the central permissible frequency (λ_{int}), and the shortest wavelength (λ_{min}) for each axon caliber. To remind the readers, for different axon calibers, we send in light at different wavelengths, ranging from 0.4 µm (chosen to avoid absorption by the proteins) to the thickness of the myelin sheath, or 1.3 µm (the upper bound of the observed biophoton wavelength), whichever is smaller for good confinement in the myelin sheath (at least 80 %). We call this upper wavelength bound the longest permissible wavelength (λ_{max}). The shortest permissible wavelength (λ_{min}) for all simulations is 0.4 µm. In addition to λ_{max}, and λ_{min}, we choose an intermediate wavelength corresponding to the central permissible frequency (mid-frequency of the permissible frequency range), denoted by λ_{int}. In a single simulation, FDTD calculates the input mode at λ_{int} and sends light at different wavelengths with the same spatial mode profile. Note that for the thinnest axons considered, λ_{max}= λ_{int}= λ_{min}=0.4 µm (d=0.4 µm, too, for good confinement).

Radius of the axon including the myelin sheath (µm)	Thickness of the myelin sheath (µm)	Longest permissible wavelength λ_{max} (µm)	Wavelength corresponding to the central permissible frequency λ_{int} (µm)	Shortest permissible wavelength λ_{min} (µm)	Percentage confinement for λ_{max}	Percentage confinement for λ_{int}	Percentage confinement for λ_{min}
1	0.40	0.40	0.40	0.40	82.13	82.13	82.13
2	0.80	0.80	0.53	0.40	82.13	91.49	95.44
3	1.20	1.20	0.60	0.40	82.30	95.47	98.28
4	1.60	1.30	0.61	0.40	87.67	97.62	99.19
5	2.00	1.30	0.61	0.40	92.17	98.68	99.58

Supplementary Figure 1. **Modal confinement in the myelin sheath.** Range of permissible wavelengths for different myelin thicknesses and the percentage of power confined in the myelin sheath for those wavelengths.

Next, we shall discuss effects of a few imperfections in detail, expanding on the points mentioned in the main text.

Continuously varying non-circular cross-sectional shape. The cross-sectional shape of an axon changes in the longitudinal direction. In our model, we twist an axon, such that it starts out with an elliptical cross-section with semi-major and semi-minor axes a and b respectively, interchanges the axes midway (25 µm) and reverts to its original shape at the end of the segment (50 µm). Since the cross-section is continuously changing, the guided modes at each section change too. An appropriate way to quantify the loss in such a structure would be to incident an eigenmode of a cylindrical waveguide (circular cross-section), and observe its transmission at the other end. Supp.

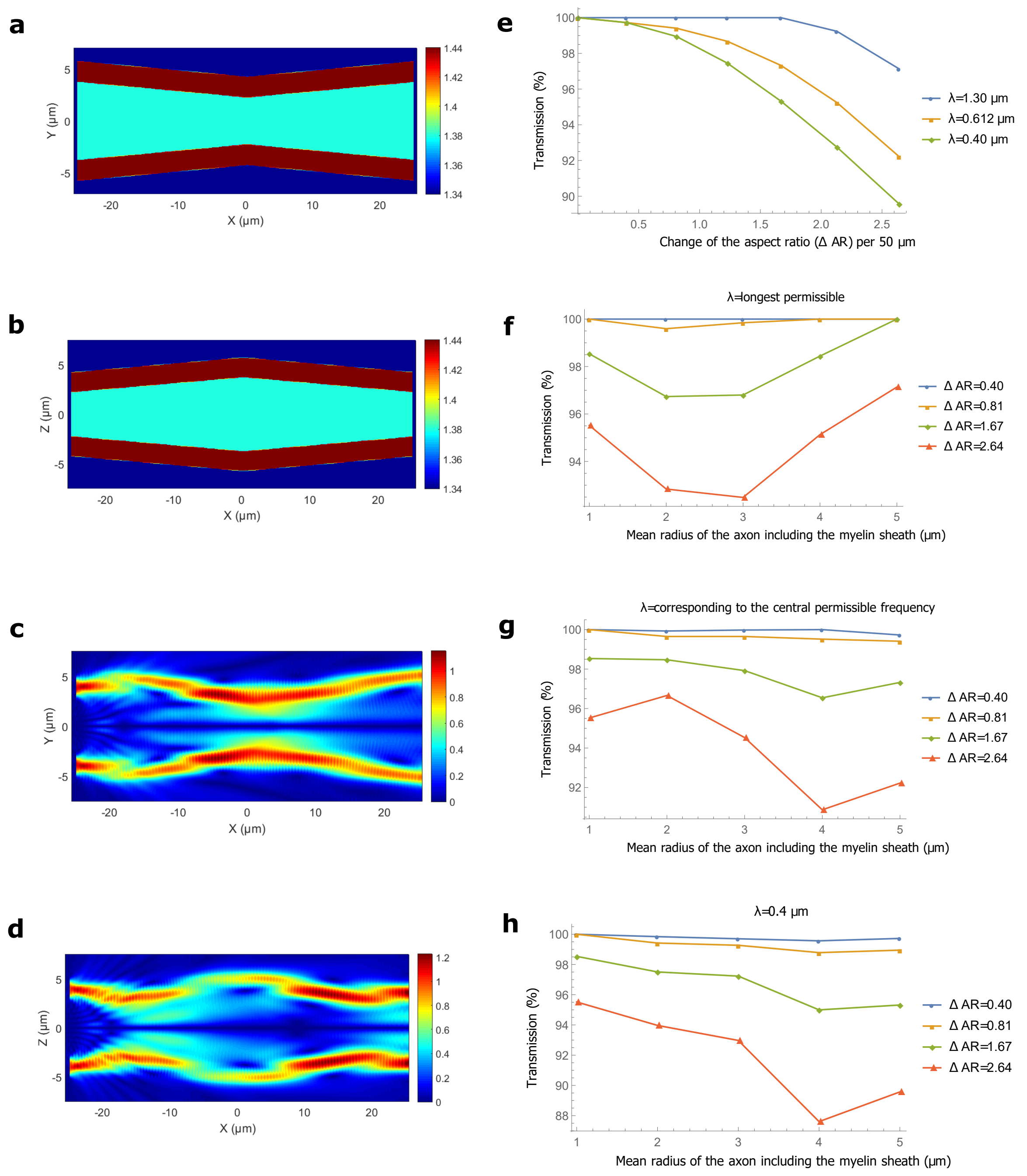

Supplementary Figure 2. **Continuously varying non-circular cross-sectional shape. (a)-(b)** The refractive index profile of a myelinated axon in the X-Y plane and the X-Z plane respectively. The semi-major and semi-minor axes of the ellipses denoting the axonal boundaries at the start of the segment are 3.75 µm, and 2.25 µm respectively (the corresponding axes for the myelin sheath's outer boundaries are 5.75 µm, and 4.25 µm respectively). **(c)-(d)** Magnitude of the electric field in the longitudinal direction (EFPL), as an eigenmode of a cylindrical waveguide ($r = 3\,\mu\text{m}$, $r' = 5\,\mu\text{m}$, and $\lambda = 1.3\,\mu\text{m}$) crosses the axonal segment in the X-Y plane and the X-Z plane respectively. **(e)** Transmission as a function of the change in the aspect ratio (ΔAR); ΔAR is defined as change in the ratio of the axes of the ellipse along two fixed orthogonal directions (here the Y and Z axes). The mean of the semi-axes of the axonal ellipse is 3 µm (corresponding mean for the myelin sheath's outer boundary is 5 µm). **(f)-(h)** Transmission as a function of the axon caliber for different wavelengths and different ΔAR.

Fig. 2a, and Supp. Fig. 2b show the longitudinal cross-section of the structure in 2 different planes (here, the X-Y and the X-Z planes). Supp. Fig. 2c–d depict the magnitude of the electric field along the length of an axon in those planes, as a cylindrically symmetric eigenmode of a cylindrical waveguide ($r = 3\,\mu m$, $r' = 5\,\mu m$, and $\lambda = 1.3\,\mu m$), identical to Fig. 1b–e from the main text, passes by. We call this EFPL (Electric Field Profile in the Longitudinal direction). Supp. Fig. 2e shows the total power transmission (calculated by integrating the real part of the Poynting vector of the output light directly across the required area, and dividing it by the source power) upto a wavelength away from the myelin sheath boundaries, as a function of the change in the aspect ratio (defined as the change in the ratio of the axes of the ellipse along two fixed orthogonal directions, here the Y and Z axes) of the ellipse per 50 µm. We notice that longer wavelengths transmit better. We see transmission as a function of axon caliber in Supp. Fig. 2f–h. Supp. Fig. 2f, dealing with transmission for the longest permissible wavelengths, shows an interesting dip in transmission for r'=2 µm, and r'=3 µm. Comparing the transmissions for certain axon caliber (e.g. r'=2 µm, and r'=3 µm), and different wavelengths in Supp. Fig. 2f–h, we observe that the intermediate wavelength has a larger transmission. We note from Supp. Fig. 1 that $\lambda_{max} = d$ for them, while for thicker myelin sheaths, i.e. r'=4 µm, and r'=5 µm, $\lambda_{max} < d$. These observations suggest that there is an intermediate wavelength somewhere between d and λ_{min} (not necessarily λ_{int}) where transmission is maximized. The propagation loss can be understood as a coupling loss between subsequent cross-sections (infinitesimally apart from each other). Shorter wavelengths have a higher number of guided modes at each cross-section than longer wavelengths, but the input mode at a shorter wavelength can get distorted more too (by exciting higher-order modes). If it is distorted beyond a certain extent, light in those higher-order modes would be lost in subsequent cross-sections that do not have similar modes. Or if these higher order modes are at a wavelength away from the myelin sheath boundaries, they are not included in the transmission. So there is a competition between the number of available modes to couple to, and the extent of distortion. An intermediate wavelength turns out to be optimum. Also, for larger ΔAR, and short wavelengths, thinner axons are better, suggesting the relevance of the absolute value of the change in the ellipse's axes. The transmission for close to ΔAR (per 50 µm) = 0.40 is close to unity for all the cases discussed. Note that the approximate equivalence of the elliptical shape and a randomly shaped cross-section for transmission of a circular mode is discussed in the Supplementary Methods.

Cross talk between axons. The neurons might be close to, or in contact with other neurons or non-neuronal cells in the brain (e.g. glia cells). Light in a myelinated axon would not leak out significantly, even if placed in direct contact with cells of lower refractive indices than the myelin sheath. However, if two or more myelinated axons are placed very close to each other (side by side), then light could leak out from one to the other. Supp. Fig. 3a shows the longitudinal refractive index profile of 2 axons ($r' = 4\,\mu m$) touching each other, and Supp. Fig. 3b is the EFPL (for those axons) when an input mode with wavelength 0.4 µm is incident on one of them. In Supp. Fig. 3c, we notice that shorter wavelengths stay confined in the myelin sheath better, as expected. Supp. Fig. 3d–f deal with transmission (see Supplementary Methods for the procedure to quantify transmission) in the myelin sheath for different axon calibers, different wavelengths, and different separation between the axons. As a general rule, axons should be a wavelength away from one another to avoid cross talk, although the confinement for the same wavelength for different axon calibers can be quite different. Multimode waveguides (greater caliber) confine light much better than those with a few modes for a particular wavelength.

For our simulations, we considered cross talk between identical axons, which is stronger than that between non-identical ones. Also, the cross-talk between axons does not imply irretrievable loss. For perfectly identical optical fibers placed in contact, it is known that there is a complete power transfer from one to the other periodically [S3]. Moreover, extrapolation of the transmission for greater axon length is not straightforward, as light could propagate in the guided modes of the composite structure (many axons touching each other), with fluctuations (or oscillations) in power from one to the other. Since the most important source of loss (more so for the smaller wavelengths) here is light leaking into the myelin sheath of a different axon (and not the inside of the axons or outside), on average the power should be divided equally among the axons touching each other, provided that the segments in contact are long enough. Extrapolation from the data in Supp. Fig.3 as an exponentiation of the fraction of the power transmitted through 50 µm should therefore be interpreted as a strict upper bound on the loss. Moreover, this might be a mechanism for information transfer between axons, leading to a collective behaviour of neurons in a nerve fiber (several axons bunched close together for a considerable length).

The power loss when the axons touch each other under different spatial orientations is significantly less. For example, when two axons cross perpendicular to each other, the power loss is less than 0.5 % for all the axon calibers.

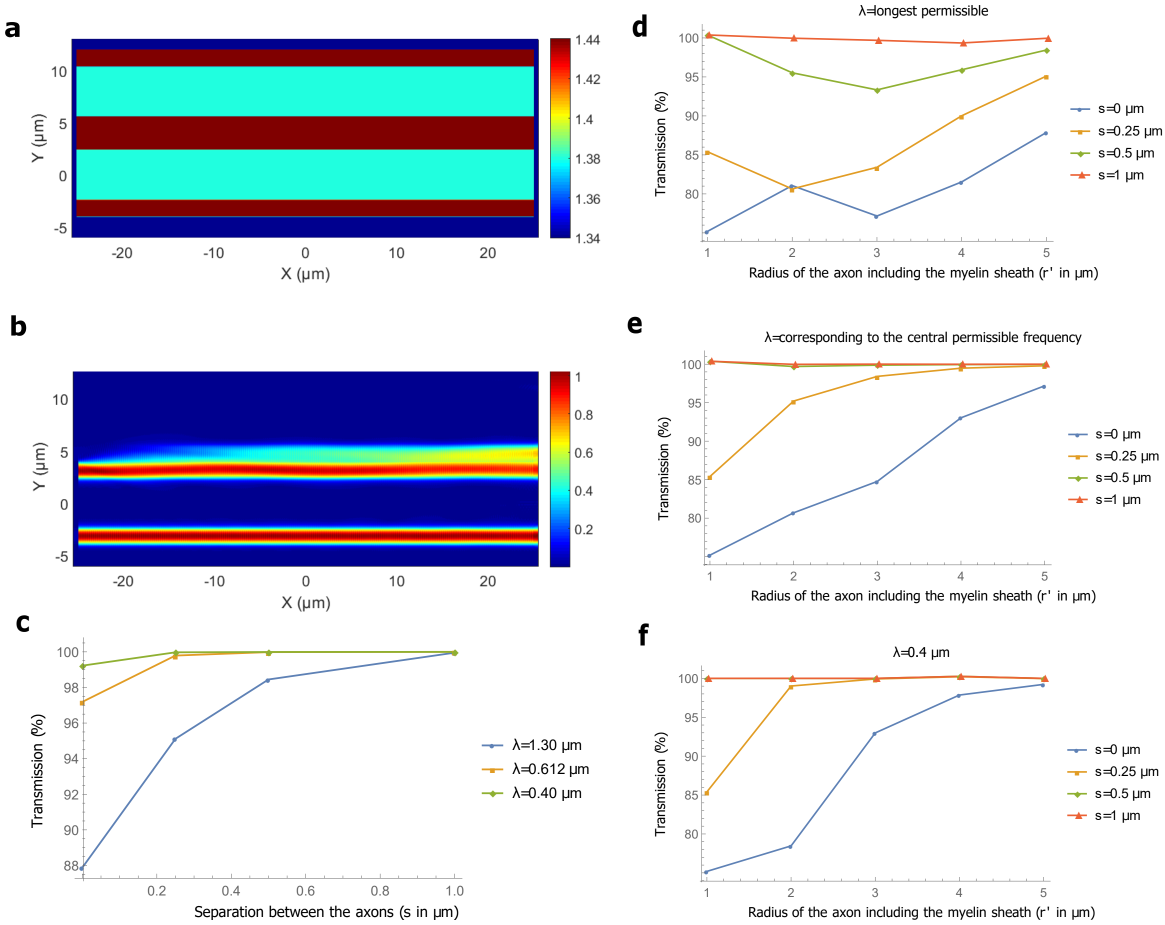

Supplementary Figure 3. **Cross talk between axons. (a)** The refractive index profile of 2 axons touching each other ($r' = 4\,\mu\text{m}$). **(b)** EFPL as the input mode with wavelength $0.612\,\mu\text{m}$ crosses the region. **(c)** Transmission as a function of the separation between axons for different wavelengths ($r' = 5\,\mu\text{m}$). **(d)-(f)** Transmission as a function of the axon caliber for different wavelengths and different separation between axons.

Guided modes inside the axon. We have taken the refractive indices of the axon, the myelin sheath, and the medium outside as 1.38, 1.44, and 1.34 respectively for almost all our simulations. A vast majority of the modes of such a waveguide are confined strongly in the myelin sheath if it is thick enough. However, a few guided modes exist which have a greater fraction of optical power inside the axon than in the myelin sheath even if the myelin sheath is thick, and the wavelength is small. This is true if the axon has a greater refractive index than the medium outside the myelin sheath, and is sufficiently thick (true if the myelin is thick and the *g-ratio* = 0.6). In the main text, we were particularly conservative and ignored the guided modes inside the axon, and treated them as loss, because we are not sure about the relevant light-guidance parameters inside the axon (see the later discussion on scatterers inside the axon). Without ignoring them, the transmission for all the inhomogeneities would be slightly better. Especially for the long paranodal regions, where some light inevitably leaks into the axon, one sees a clear difference.

Nodal and paranodal regions with inclusion of the guided modes inside the axon. Let's be optimistic and assume that the inside of the axon is homogeneous (has a constant refractive index of 1.38) to obtain an upper limit on the transmission as light crosses the nodal and paranodal regions. In Supp. Fig. 4, we plot the modal transmission (power transmission in all the guided modes of the myelinated axon) after two paranodes and a node in between. We shall call two paranodes with a node in between a PNP (Paranode-Node-Paranode) region. We notice that for *p-ratio* = 2.5, almost all the light for different axon calibers stays in the guided modes within a wavelength span from the myelin sheath (comparing it with Fig. 2c–f in the main text, where we took the transmission in the guided modes only upto a wavelength away from the myelin sheath boundaries). Also, for longer paranodal regions, the smaller wavelengths scatter more into the axon (and also in the medium outside the myelin sheath) than the longer wavelengths, as is evident from the difference in the transmission as compared to Fig. 2c–f in the main text. As

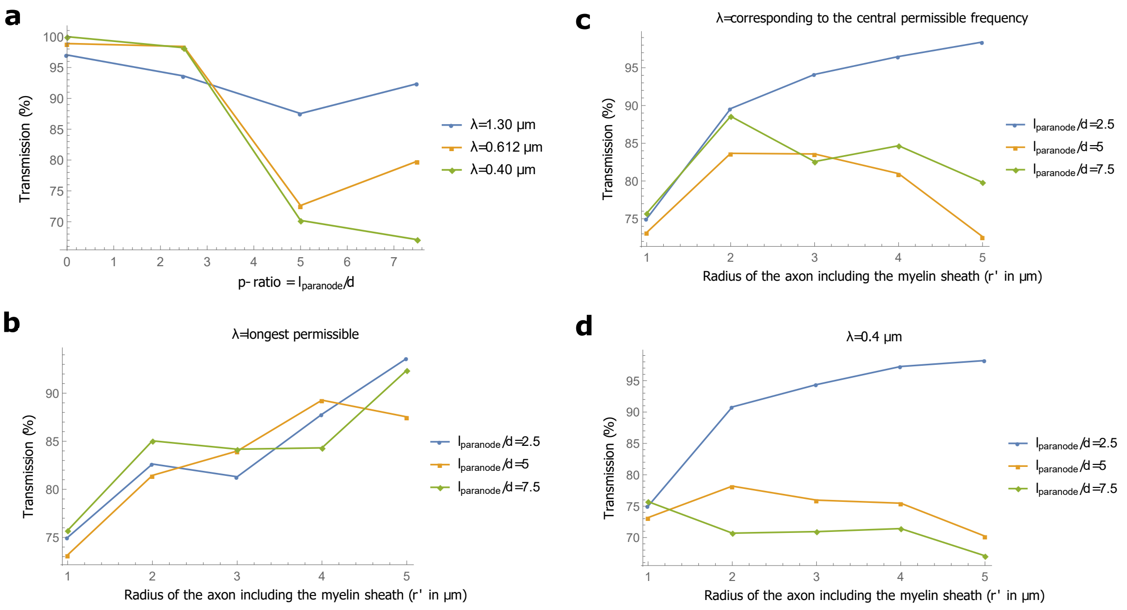

Supplementary Figure 4. **Nodal and paranodal region with inclusion of the guided modes inside the axon.** **(a)** Transmission in all the guided modes as a function of $l_{paranode}/d$ (*p-ratio*), where $l_{paranode}$ is the length of a paranode, and d is the thickness of the myelin sheath. The *p-ratio* is varied by changing the length of the paranode, keeping the axon caliber constant ($r = 3\,\mu$m, and $r' = 5\,\mu$m). **(b)-(d)** Transmission in the guided modes as a function of the axon caliber for different wavelengths and different *p-ratios*.

an example, the transmission in all the guided modes for $\lambda = 0.4\,\mu$m and $r' = 5\,\mu$m is 67.09 %, but that within a wavelength span of the myelin sheath is only 33.78 %. In a realistic scenario where there are scatterers inside the axon, the transmission would lie between these values. So, the plots in Supp. Fig. 4 should be interpreted as an upper bound on the transmission and Fig. 2c–f in the main text should be interpreted as a lower bound.

Subsequent nodal and paranodal regions. If the inhomogeneities in the rest of the internodal length is within the acceptable values, there would be no more loss as the rest of the light is in the guided modes. However, since there is mixing of modes as light passes through the paranodal regions, one might wonder how the mixture of modes behaves as it encounters the next PNP region (after an internodal length). Supp. Fig. 5 shows the transmission in the guided modes after subsequent PNP regions for different axon calibers and different wavelengths for *p-ratio* = 2.5. Note that the transmission is re-normalized to unity after each PNP region, such that the total modal transmission after 3 PNP regions is the product of the modal transmission after each of these regions. In general, the longest permissible wavelengths (weakly confined) get better or almost saturate after 3 PNP regions. For shorter wavelengths, the modal transmission after each segment is less predictable since they are more prone to distortions in the shape of the myelin and undergo significant mode mixing. However, for most of the cases, the modal transmission fluctuates both ways (increases and decreases), and an average close to the first pass is approximately true. Thus, we can approximately predict the modal transmission after multiple PNP regions by exponentiating the modal transmission through one.

Effect of the scatterers and possibility of light guidance inside the axon. There are many potential scatterers inside the axon, e.g. microtubules, mitochondria, agranular endoplasmic reticulum, and multivesicular bodies. We would not only need the refractive indices of these structures, but also their shapes, sizes and spatial distribution, to accurately predict their effect on light guidance. We have little relevant (and sometimes conflicting) data. For instance, Sato et al. measured the refractive index of microtubules to be 1.512 [S4], but Mershin et al. measured the refractive index of tubulin, the building block of microtubules to be 2.9 [S5]. Microtubules are one of the most numerous structures inside the axon, forming the cytoskeleton and a rail-road for the transport of materials inside the axon. The density of microtubules varies during the axon differentiation from ∼1 % in the initial phase to ∼3 % during the most dense phase and again drops (to a value we do not know) [S6].

To study the scattering effects of the microtubules on our previous simulations, we distribute them randomly (but according to a uniform distribution) such that they occupy ∼2 % of the volume inside the axon. Their refractive index is taken to be 1.5 and they are placed in a medium of refractive index 1.38. We had seen that in a few of our

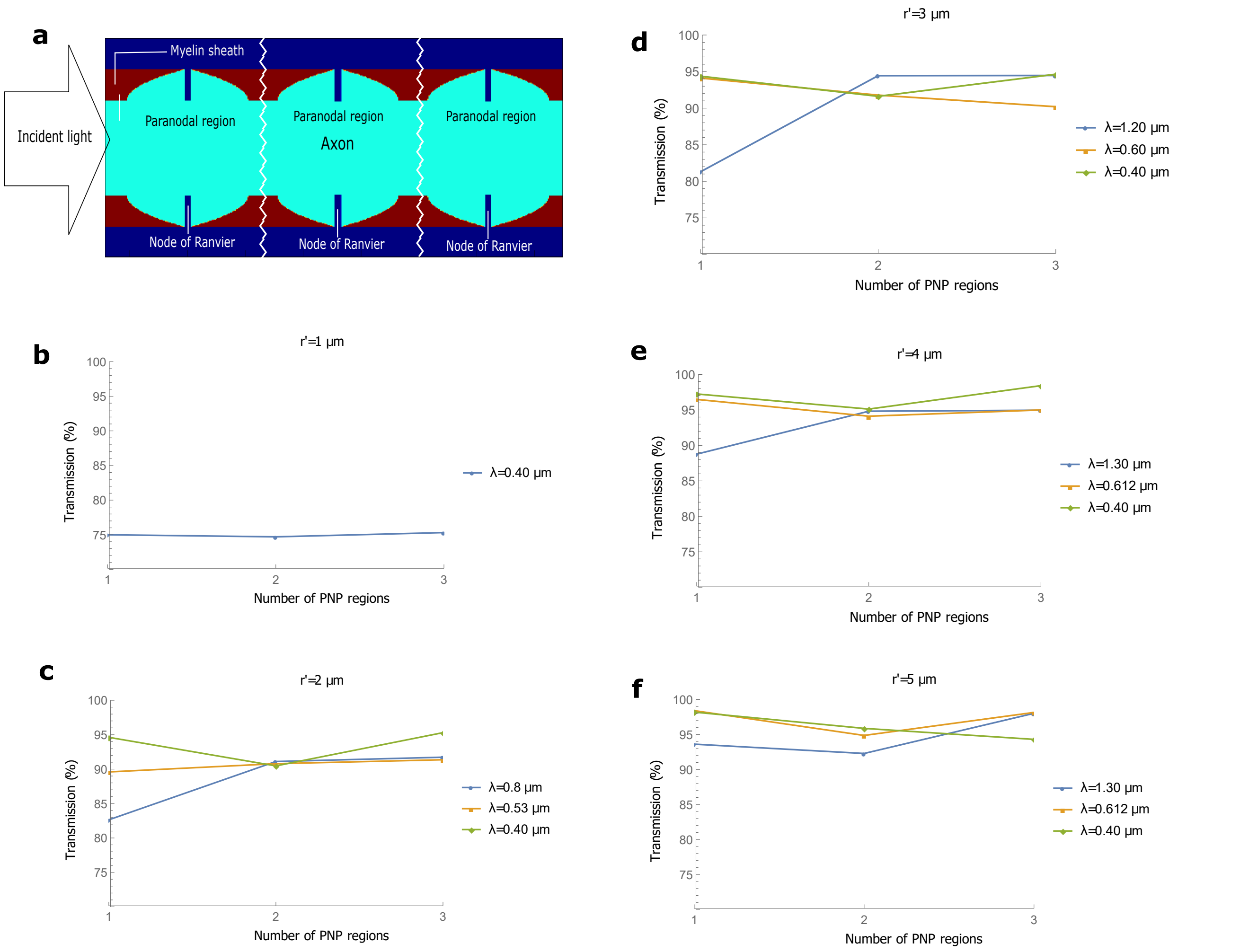

Supplementary Figure 5. **Subsequent PNP regions. (a)** Geometry of 3 PNP regions placed sequentially. A PNP (Paranode-Node-Paranode) region is defined as two facing paranodes with a node in between. The discontinuity between two PNP regions implies the presence of a straight and uniform internode there. **(b)-(f)** Transmission in all the guided modes as a function of the number of PNP regions for different wavelengths and different axon calibers. Note that the transmission is normalized to unity after each PNP region, such that the total modal transmission after 3 PNP regions is the product of the modal transmission after each of these regions.

previous simulations, some fraction of optical power leaked into the axon, e.g. for large variation in the cross-sectional area, and paranodal regions. We ran the simulations again, this time in the presence of the microtubules. We found negligible variation in the transmission, both inside and outside the axon ($\pm \sim 1$ %). Even the light that leaked into the axon did not scatter much in the presence of the microtubules (owing to their small size and close to uniform distribution).

There are proposals of light guidance by the microtubules and mitochondria inside the axon [S7–S9]. But they are too tiny for this to be realistic in the observed biophotonic wavelength range. Mitochondria are typically less than a few microns long, and microtubules are too thin (tubular structures with the inner and outer diameters as $\sim$12 nm and $\sim$24 nm respectively) to confine light in the biophotonic wavelength range (waveguide dimension should be comparable to the wavelength of light). However, if we assume that the microtubules are uniformly distributed, we can approximately average the refractive index of the composite system comprising of the axonal fluid and microtubules as $\sqrt{f \times n_m^2 + (1-f) \times 1.34^2}$, where f and n_m are the volume fraction and the refractive index of the microtubules respectively, and 1.34 is the refractive index of the fluid inside the axon. The average is possible since the microtubules are much smaller than the wavelength of light, and so is the average separation between them [S6]. We could wonder whether this composite system can guide light, which is only possible if the inside of the axon has a higher refractive index than the medium outside. If the refractive index of the microtubules is 1.5, then a typical volume fraction, e.g.

1.7 % would give $n_{avg} = 1.343$, and if the refractive index is 2.9, then $n_{avg} = 1.381$. Since the observed refractive indices inside the axon in both the longitudinal and the transverse directions are in a broad range (1.34–1.38 in [S10], and 1.35–1.40 in [S11]), assuming the axon as a uniform medium with refractive index 1.38 is not entirely correct. Moreover, the axons can be in direct contact with glia cells which can have comparable refractive indices as the inside of the axon. This would prevent guided modes to exist inside the axon. Note that if the refractive index of the axon is lower than 1.38, most of our simulations in the main text will yield slightly better transmission as the light guidance mainly depends on the refractive index contrast. And if the refractive index of the outside is greater than 1.34, the transmission will suffer slightly. However, since the refractive index of the myelin sheath is much larger than both the regions, these effects would not be too significant for most of the simulations.

However, if we assume that the axons are not in contact with other glia cells, and have a higher refractive index than the interstitial fluid outside, then weak guidance might still be possible if the mode does not scatter off of the bigger (but less numerous) scatterers (e.g. mitochondria, Endoplasmic Reticulum, and vesicles). We do not know the volume fraction of these scatterers precisely but some work, e.g. [S12] suggest that they occupy at least 10 % of the volume. We model these scatterers as ellipsoids with the 3 semi-minor axes ranging from 0.1 µm to 0.4 µm, 0.1 µm to 0.4 µm, and 1 µm to 3 µm respectively and place them in axon with $r' = 5\,\mu m$. Their refractive indices are taken to be 1.4. Let's take 2 different values of the refractive index of the axon. For a value 1.38, the total power transmission (calculated by integrating the real part of the Poynting vector of the output light directly across the required area, and dividing it by the source power) upto a wavelength away from the axonal boundaries in a 100 µm long structure for a mode confined inside the axon at wavelength 0.612 µm is 75.47 %, while for 1.3 µm wavelength, the transmission is 95.93 %. If the axon's refractive index is 1.35, then the transmission for the wavelength 0.612 µm is 16.06 %, while no guided modes exist for the wavelength 1.3 µm. A lower density of these scatterers, or smaller sizes, (or larger wavelengths than 0.612 µm) would, of course yield greater transmission. The transmission for 0.612 µm wavelength light is different for different refractive index values of the axon because scattering depends strongly on the refractive index contrast. A mitochondrion (refractive index 1.40) placed in a medium with refractive index 1.35 would act as a much stronger scatterer than if placed in a medium with refractive 1.38. Thus, an average uniform refractive index of 1.38 for the axon might still guide light at large wavelengths, but an average uniform refractive index of 1.35 seems more believable (assuming the refractive index of microtubules to be ∼1.50). In this case, the bigger scatterers lead to significant loss, even if the microtubules themselves do not. Therefore, we do not believe that there could be guided modes inside the axon which can transmit efficiently.

We again ran many of our previous simulations (with the input mode confined primarily in the myelin sheath) in the presence of all these scatterers inside the axon. We varied the refractive index of the axon from 1.34 (the refractive index of the medium outside) to 1.38. We verified that light well confined in the myelin sheath does not see these scatterers at all. Even when the light diverges into the axon because of the geometry of the structure (e.g. the varying cross-sectional area), there is still not a dramatic variation in the transmission. Both the transmission in the myelin sheath up to a wavelength away from the boundaries, and the total transmission across the whole cross-section (including the inside of the axon) do not change greatly; the observed variation was on the order of a few percent. Note that for a few simulations, ∼15–20 % of the fraction of output light can be inside the axon. The light diverging inside need not even be in the guided modes of the waveguide. This runs counter to intuition, since we saw that a guided mode inside the axon scattered badly. This unintuitive phenomenon can be explained again by the unconventional nature of this waveguide, where all the light leaking inside is not irretrievably lost (even if it is not in the guided modes of the structure). It can come back to the myelin sheath without interacting strongly with the scatterers. This shows that we might have been too conservative while considering the power only within a wavelength of the myelin sheath boundaries. However, there still might be other phenomena happening (e.g. absorption) inside the axon, and we prefer to be cautious about the inside.

Next, we shall see how varying the refractive index of the axon affects the transmission of a mode (confined primarily in the myelin sheath) in the PNP region.

Varying the refractive indices of the axon and the cytoplasmic loops. We have observed that the paranodal regions might be the main contributor to loss (if the other inhomogeneities are low). For our simulations so far, we have assumed that the refractive index of the cytoplasmic loops is the same as that of the axon (1.38). As far as we know, no direct measurement of the refractive index of these loops has been performed, but they are however considered *'dense'* [S13].Since these loops are part of glia cells, which usually have higher refractive indices, these loops might have higher refractive indices than the inside of the axon too. In Supp. Fig. 6, we show the result of

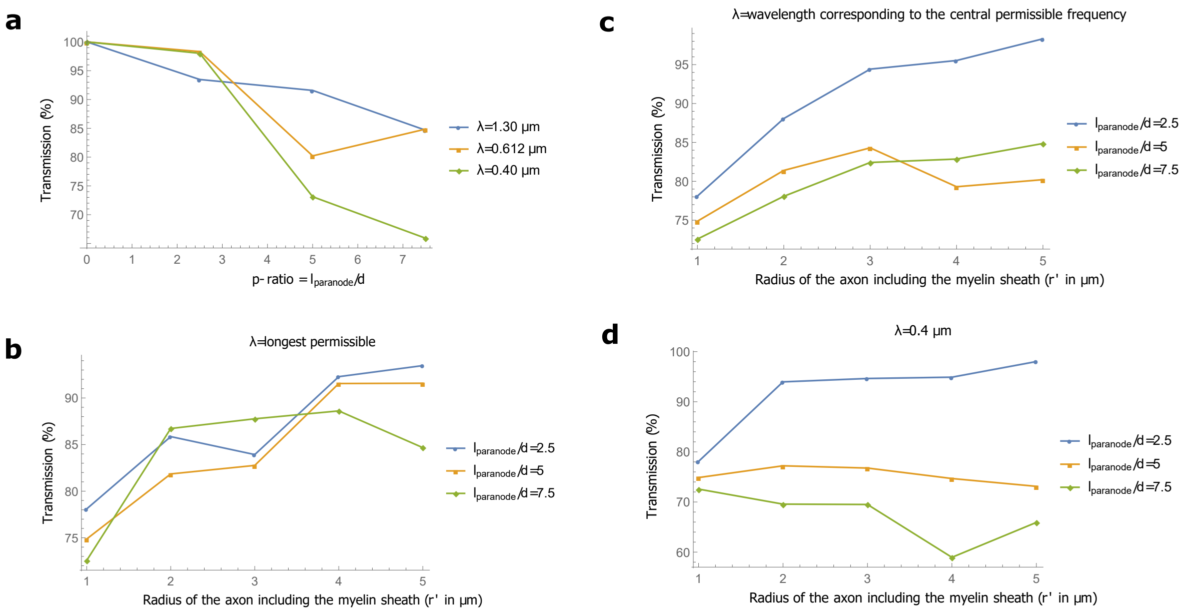

Supplementary Figure 6. **Nodal and paranodal regions for a different set of refractive indices.** The refractive indices of the axon and the cytoplasmic loops are taken as 1.34 and 1.38 respectively. **(a)** Transmission in all the guided modes as a function of the *p-ratio* for different wavelengths ($r' = 5\,\mu\text{m}$). **(b)-(d)** Transmission in the guided modes as a function of the axon caliber for different wavelengths and different paranodal lengths.

another set of simulations where the refractive index of the axon is kept the same as the medium outside (1.34), and that of the loops is higher (1.38). We find that in almost all the cases (different paranodal lengths, different wavelengths, and different axon calibers), the transmission in the guided modes is higher as compared to the previous set of simulations (see Fig. 2c–f in the main text). If cytoplasmic loops have a higher refractive index, then they prevent the mode from diverging into the axon, and serve as weak waveguides themselves. Note that for the same refractive index of the axon and the cytoplasmic loops (e.g. 1.35 each), the results would be similar to those when both had their refractive indices 1.38.

Mathematics of mode expansion and transmission calculation. We have often mentioned the expansion of the output field in the basis of guided modes, and the calculation of the transmission by evaluating the Poynting vector, integrating its real part across the area of interest and dividing it by the input power. Here we give the mathematics of these procedures.

Let the electric (E) and magnetic (M) field profiles (frequency domain) of the light incident in the axon be denoted by $\vec{E}_{in}$, and $\vec{H}_{in}$ respectively, and the field profiles of the light at the terminal end of the axon segment in the transverse plane (perpendicular to the length) be denoted by $\vec{E}_{out}$, and $\vec{H}_{out}$ respectively. We can express

$$\begin{aligned}\vec{E}_{out} &= \vec{E}_{guided} + \vec{E}_{non-guided} \\ \vec{H}_{out} &= \vec{H}_{guided} + \vec{H}_{non-guided}\end{aligned} \tag{S1}$$

where $\vec{E}_{guided}$, and $\vec{H}_{guided}$ are the fields for the fraction of light in the finite number of guided modes of the waveguide, and $\vec{E}_{non-guided}$, and $\vec{H}_{non-guided}$ are the fields for the fraction in the infinite number of non-guided modes. Light in the non-guided modes of a uniform structure would be lost eventually. The guided part can further be expanded as

$$\begin{aligned}\vec{E}_{guided} &= \sum_i (a_i \vec{E}_i^{forward} + b_i \vec{E}_i^{backward}) \\ \vec{H}_{guided} &= \sum_i (a_i \vec{H}_i^{forward} - b_i \vec{H}_i^{backward})\end{aligned} \tag{S2}$$

where $\vec{E}_i$, and $\vec{H}_i$ are the fields corresponding to a guided mode ϕ_i, and a_i and b_i are the transmission coefficients for the forward and backward propagating waves respectively. The summation is over the entire set of the orthogonal

guided modes of the structure. The coefficients are given in terms of the overlap integrals as

$$a_i = 0.25 \times \left(\frac{\int (\overrightarrow{E}_{guided} \times \overrightarrow{H}_i^*) \cdot \overrightarrow{dS}}{P_i} + \frac{\int (\overrightarrow{E}_{guided}^* \times \overrightarrow{H}_i) \cdot \overrightarrow{dS}}{P_i^*}\right)$$
$$b_i = 0.25 \times \left(\frac{\int (\overrightarrow{E}_{guided} \times \overrightarrow{H}_i^*) \cdot \overrightarrow{dS}}{P_i} - \frac{\int (\overrightarrow{E}_{guided}^* \times \overrightarrow{H}_i) \cdot \overrightarrow{dS}}{P_i^*}\right) \tag{S3}$$

where $\overrightarrow{dS}$ is the differential area element in the transverse plane of interest, and the complex power of the ith mode ϕ_i is

$$P_i = 0.5 \times \int (\overrightarrow{E}_i \times \overrightarrow{H}_i^*) \cdot \overrightarrow{dS} \tag{S4}$$

The percentage transmission into all the guided modes of the structure is given by

$$T = \frac{0.5 \times \int Re(\overrightarrow{E}_{guided} \times \overrightarrow{H}_{guided}^*) \cdot \overrightarrow{dS}}{0.5 \times \int Re(\overrightarrow{E}_{in} \times \overrightarrow{H}_{in}^*) \cdot \overrightarrow{dS}} \times 100 \tag{S5}$$

Here, $\overrightarrow{S}_{guided} = \overrightarrow{E}_{guided} \times \overrightarrow{H}_{guided}^*$ is the time averaged Poynting vector for the guided fraction of the output light, and $Re()$ denotes the real part. Integration of the real part of the Poynting vector across an area quantifies the time-averaged power flow through that area, while the integration of the imaginary part quantifies the reactive power (e.g. because of interference due to a standing wave).

In specific contexts (in particular after the PNP regions, see Fig. 2c–f in the main text), we integrate the real part of the Poynting vector (with the electromagnetic fields corresponding to the guided portion of the output light) across the myelin sheath up to a wavelength away from the boundaries to obtain the percentage transmission

$$T = \frac{0.5 \times \int_{\rho=r-\lambda}^{\rho=r+\lambda} Re(\overrightarrow{E}_{guided} \times \overrightarrow{H}_{guided}^*) \cdot \overrightarrow{dS}}{0.5 \times \int Re(\overrightarrow{E}_{in} \times \overrightarrow{H}_{in}^*) \cdot \overrightarrow{dS}} \times 100 \tag{S6}$$

where ρ is the radial coordinate, λ is the wavelength, and r and r' are the inner and outer radius of the myelin sheath as defined earlier. We include only the guided fraction of the light because the non-guided fraction is expected to decay over the course of the long internode following the PNP region (provided that the internode is approximately uniform).

In certain other instances (e.g. varying cross-sectional area and shape), where the cross-section continuously changes, some fraction of light in the non-guided modes at a particular cross-section might be included in the the basis of guided modes at an adjoining cross-section and vice-versa. Therefore, it is more appropriate to observe the total power transmission (up to a wavelength of the myelin sheath boundaries) instead of the modal transmission. In such cases we integrate the real part of the Poynting vector with the fields corresponding to the output light directly to obtain the percentage transmission

$$T = \frac{0.5 \times \int_{\rho=r-\lambda}^{\rho=r+\lambda} Re(\overrightarrow{E}_{out} \times \overrightarrow{H}_{out}^*) \cdot \overrightarrow{dS}}{0.5 \times \int Re(\overrightarrow{E}_{in} \times \overrightarrow{H}_{in}^*) \cdot \overrightarrow{dS}} \times 100 \tag{S7}$$

Supplementary Methods

Continuously varying non-circular cross-sectional shape. We simulate the effect of the change in the cross-sectional shape of an axon in the longitudinal direction by twisting an elliptical axon. The semi-major and the semi-minor axes of the ellipse (a and b resp.) at x = 0 (the starting point of the axon) are changed for different simulations. We incident an eigenmode of a circular axon with $r = (a+b)/2$, and $r/r' = 0.6$. The myelin sheath boundary is another ellipse with its axes, $a' = a+d$ and $b' = b+d$, where $d = r' - r$. The myelin sheath is thus an approximate parallel curve to the axon. The shape of the axon changes continuously such that at one-fourth of the axonal segment, it becomes a perfect circle with radius $r = (a+b)/2$, at half the length, it interchanges its axes, and at the end of the segment (50 µm), it resumes its original shape. The area of the cross-section remains almost constant by this twist (less than 10 % variation for all the simulations). Different values of the change in the aspect ratio (ΔAR) are obtained by adopting the same procedure for ellipses with different semi-axes.

An approximate equivalence between an elliptical shape and a random cross-sectional shape (as in the main text) can be established. The equation of an ellipse in polar coordinates is $\rho(\theta) = ab/(\sqrt{(b\cos\theta)^2 + (a\sin\theta)^2})$, where ρ is the radial coordinate and θ is the polar angle from the a axis. The mean of the distance of the points from the origin is very close to $r = (a+b)/2$ (less than 7 % variation for all the simulations). In the main text, we generated random points according to a Gaussian distribution along the circumference of the cross-section, and the s.d. of the separation of those points from a circle of radius r is taken as the degree of inhomogeneity. For an ellipse, the s.d. of separation from a circle of radius $r = (a+b)/2$ can similarly be calculated as $\sqrt{1/(2\pi)\int_0^{2\pi}((a+b)/2 - ab/(\sqrt{(b\cos\theta)^2 + (a\sin\theta)^2}))^2 d\theta}$. We compare transmission in an elliptic (non changing cross-sectional area) waveguide, and a waveguide with an arbitrary cross-sectional area with the same s.d for some of the simulations, and find that there is comparable or higher loss in an elliptical waveguide. This suggests that an axon with changing cross sectional shape (random) along its length might also undergo similar loss as a twisting elliptical axon. We quantify the change in aspect ratio (ΔAR) as a measure of the change in the cross-sectional shape for elliptical shapes. For example, if the cross-section is an ellipse with $a = 3.9\,\mu$m, and $b = 2.1\,\mu$m at x = 0, after the twisting procedure, $\Delta AR = 2 \times (3.9/2.1 - 2.1/3.9) = 2.64$ (the factor 2 shows that it is twisted to get back to the original shape after the segment).

The transmission is calculated by integrating the real part of the Poynting vector across an area between 2 ellipses, one with the semi-axes $a + d + \lambda$, $b + d + \lambda$, and the other with the semi-axes, $a - \lambda$, and $b - \lambda$, where λ is the wavelength of the light, and the other symbols hold their previous meanings. The procedure adopted to account for the change in the mode profiles with wavelength is the same as discussed in the Methods of the main text (e.g. as in bends). We divide the transmission for the larger wavelengths by the transmission within a wavelength of the myelin sheath for a circular waveguide on sending in a mode with the central permissible frequency to obtain the normalised transmission. The losses are in fact a combination of the insertion loss (coupling loss of the input light to the first cross section it sees) and the propagation loss (can be understood as coupling losses for subsequent cross-sections), but as a conservative approach, we allocate everything to the propagation loss. Under this assumption, we expect that an ellipse with a larger (or smaller) aspect ratio (a/b) to start with, would have almost similar transmission if ΔAR is the same (for the same mean caliber r, i.e. $(a+b)/2$). For a waveguide with arbitrary cross-sectional shape that changes continuously, an analogous picture (to the twisting of an elliptical waveguide) is to start with some random shape, then reduce the randomness to reach a perfect circular shape, then increase the randomness again to arrive at a shape with the axes reversed (a $\pi/2$ rotated form of the original shape), and carry out this procedure again to arrive at the original shape at the end of 50 µm.

Cross-talk between axons. We place two identical axons side by side, send in light through one of them and note the power (by integrating the real part of the Poynting vector across the myelin sheath only) transmitted across the same axon in which the mode was incident. We divide the power for each wavelength by the power transmitted in the myelin sheath alone (not up to a wavelength) in the absence of the second axon to obtain the normalised transmission.

[S1] Kawakami, S., & Nishida, S. Characteristics of a doubly clad optical fiber with a low-index inner cladding. *IEEE J. Quantum Electron.* **10**, 879-887 (1974).
[S2] Sairam, K. V. S. S. S. S. *Optical Communications* (Laxmi Publications Ltd., 2007).
[S3] Snyder, A. W., & Love, J. *Optical Waveguide Theory* (Chapman and Hall, Ltd., 1983).
[S4] Sato, H., Ellis, & G. W., Inoué, S. Microtubular origin of mitotic spindle form birefringence. Demonstration of the applicability of Wiener's equation. *J. Cell Biol.* **67**, 501-517 (1975).
[S5] Mershin, A., Kolomenski, A. A., Schuessler, H. A., & Nanopoulos, D. V. Tubulin dipole moment, dielectric constant and quantum behavior: computer simulations, experimental results and suggestions. *Biosyst.* **77**, 73-85 (2004).
[S6] Yu, W., & Baas, P. W. Changes in microtubule number and length during axon differentiation. *J. Neurosci.* **14**, 2818-2829 (1994).
[S7] Thar, R., & Kühl, M. Propagation of electromagnetic radiation in mitochondria? *J. Theor. Biol.* **230**, 261-270 (2004).
[S8] Rahnama, M. et al. Emission of mitochondrial biophotons and their effect on electrical activity of membrane via microtubules. *J. Integr. Neurosci.* **10**, 65-88 (2011).
[S9] Jibu, M., Hagan, S., Hameroff, S. R., Pribram K. H., & Yasue K. Quantum optical coherence in cytoskeletal microtubules: implications for brain function. *Biosystems* **32**, 195-209 (1994).
[S10] Antonov, I.P. et al. Measurement of the radial distribution of the refractive index of the Schwanns sheath and the axon of a myelinated nerve fiber in vivo. *J. Appl. Spectrosc.* **39**, 822-824 (1983).
[S11] Wang, Z. et al. Topography and refractometry of nanostructures using spatial light interference microscopy. *Opt. Lett.*, **35** 208210 (2010).
[S12] Zelena, J. Bidirectional movements of mitochondria along axons of an isolated nerve segment. *Z. Zellforsch.*, **92** 186196 (1968).
[S13] Chang, D. C., Tasaki, I., Adelman, W. J., & Leuchtag, H. R. *Structure and Function in Excitable Cells* (Plenum Press, 1983).
